# Supplementary material for: Modeling glioblastoma heterogeneity as a dynamic network of cell states
Source: Mol Syst Biol. 2021 Sep 16;17(9):e10105. doi: 10.15252/msb.202010105 (PMC8444284; doi:10.15252/msb.202010105)
Supplement: Supplementary file 5 — Source Data for Figure 3 [file MSB-17-e10105-s001.zip › Figure3A_sourcedata/GSEA_3065/nosigdb_state5.GseaPreranked.1622718435914/LARSSON_STATE_5.html]

Details for gene set LARSSON\_STATE\_5[GSEA]

|  || Dataset | state5 |
| Phenotype | NoPhenotypeAvailable |
| Upregulated in class | na\_pos |
| GeneSet | LARSSON\_STATE\_5 |
| Enrichment Score (ES) | 0.43880463 |
| Normalized Enrichment Score (NES) | 1.7708628 |
| Nominal p-value | 0.0 |
| FDR q-value | 0.012395338 |
| FWER p-Value | 0.1 |
Table: GSEA Results Summary

  

Fig 1: Enrichment plot: LARSSON\_STATE\_5      
 Profile of the Running ES Score & Positions of GeneSet Members on the Rank Ordered List

  

| PROBE | GENE SYMBOL | GENE\_TITLE | RANK IN GENE LIST | RANK METRIC SCORE | RUNNING ES | CORE ENRICHMENT || 1 | SNTG1 |  |  | 4 | 0.988 | 0.0184 | Yes |
| 2 | VMP1 |  |  | 5 | 0.967 | 0.0368 | Yes |
| 3 | KCNQ1OT1 |  |  | 8 | 0.931 | 0.0543 | Yes |
| 4 | CD9 |  |  | 14 | 0.857 | 0.0701 | Yes |
| 5 | MDM4 |  |  | 21 | 0.811 | 0.0849 | Yes |
| 6 | APLP2 |  |  | 31 | 0.733 | 0.0978 | Yes |
| 7 | PRH1 |  |  | 35 | 0.718 | 0.1112 | Yes |
| 8 | APP |  |  | 38 | 0.712 | 0.1245 | Yes |
| 9 | SQLE |  |  | 39 | 0.705 | 0.1380 | Yes |
| 10 | ADGRV1 |  |  | 40 | 0.703 | 0.1514 | Yes |
| 11 | NKTR |  |  | 42 | 0.700 | 0.1646 | Yes |
| 12 | SPCS2 |  |  | 44 | 0.689 | 0.1776 | Yes |
| 13 | FUS |  |  | 47 | 0.665 | 0.1900 | Yes |
| 14 | LAPTM4A |  |  | 51 | 0.641 | 0.2019 | Yes |
| 15 | CANX |  |  | 53 | 0.634 | 0.2139 | Yes |
| 16 | B2M |  |  | 61 | 0.601 | 0.2246 | Yes |
| 17 | HMGCR |  |  | 79 | 0.562 | 0.2334 | Yes |
| 18 | LENG8 |  |  | 89 | 0.543 | 0.2427 | Yes |
| 19 | CD46 |  |  | 104 | 0.523 | 0.2511 | Yes |
| 20 | TIMP1 |  |  | 109 | 0.518 | 0.2605 | Yes |
| 21 | PDIA4 |  |  | 112 | 0.514 | 0.2701 | Yes |
| 22 | TMED9 |  |  | 119 | 0.508 | 0.2791 | Yes |
| 23 | TFPI2 |  |  | 124 | 0.504 | 0.2882 | Yes |
| 24 | BCAP29 |  |  | 127 | 0.502 | 0.2976 | Yes |
| 25 | IGFBP2 |  |  | 137 | 0.491 | 0.3059 | Yes |
| 26 | RRBP1 |  |  | 147 | 0.486 | 0.3142 | Yes |
| 27 | PNISR |  |  | 150 | 0.482 | 0.3231 | Yes |
| 28 | TMEM59 |  |  | 151 | 0.481 | 0.3323 | Yes |
| 29 | DNM1 |  |  | 152 | 0.480 | 0.3415 | Yes |
| 30 | DYNC1H1 |  |  | 160 | 0.475 | 0.3497 | Yes |
| 31 | GGA1 |  |  | 164 | 0.473 | 0.3584 | Yes |
| 32 | MCAM |  |  | 174 | 0.460 | 0.3661 | Yes |
| 33 | CTGF |  |  | 199 | 0.440 | 0.3718 | Yes |
| 34 | CHST12 |  |  | 206 | 0.436 | 0.3795 | Yes |
| 35 | SYPL1 |  |  | 231 | 0.418 | 0.3847 | Yes |
| 36 | RPN2 |  |  | 244 | 0.411 | 0.3912 | Yes |
| 37 | PTPRG |  |  | 245 | 0.411 | 0.3991 | Yes |
| 38 | TMX1 |  |  | 255 | 0.406 | 0.4058 | Yes |
| 39 | HELLS |  |  | 280 | 0.393 | 0.4106 | Yes |
| 40 | CPD |  |  | 317 | 0.374 | 0.4136 | Yes |
| 41 | FAF2 |  |  | 345 | 0.363 | 0.4175 | Yes |
| 42 | LGALS3BP |  |  | 349 | 0.361 | 0.4241 | Yes |
| 43 | RPN1 |  |  | 351 | 0.360 | 0.4308 | Yes |
| 44 | KDELR1 |  |  | 366 | 0.355 | 0.4360 | Yes |
| 45 | ADAM9 |  |  | 421 | 0.334 | 0.4363 | Yes |
| 46 | CLN5 |  |  | 497 | 0.312 | 0.4338 | Yes |
| 47 | EPRS |  |  | 506 | 0.309 | 0.4388 | Yes |
| 48 | TEX264 |  |  | 620 | 0.281 | 0.4314 | No |
| 49 | TECR |  |  | 633 | 0.278 | 0.4354 | No |
| 50 | DKK1 |  |  | 1388 | 0.163 | 0.3536 | No |
| 51 | SRPX |  |  | 1409 | 0.161 | 0.3545 | No |
| 52 | CCND2 |  |  | 4539 | -0.016 | 0.0026 | No |
| 53 | CENPE |  |  | 4787 | -0.025 | -0.0247 | No |
| 54 | BTG1 |  |  | 5087 | -0.037 | -0.0576 | No |
| 55 | SMC4 |  |  | 6517 | -0.102 | -0.2165 | No |
| 56 | HMOX1 |  |  | 6561 | -0.106 | -0.2193 | No |
| 57 | ZMAT3 |  |  | 6960 | -0.134 | -0.2616 | No |
| 58 | CCK |  |  | 7003 | -0.137 | -0.2637 | No |
| 59 | GABBR2 |  |  | 7041 | -0.141 | -0.2652 | No |
| 60 | TSHZ1 |  |  | 7088 | -0.144 | -0.2676 | No |
| 61 | PHLDA3 |  |  | 7307 | -0.168 | -0.2889 | No |
| 62 | SARS |  |  | 7503 | -0.191 | -0.3072 | No |
| 63 | GADD45A |  |  | 7633 | -0.208 | -0.3178 | No |
| 64 | PCBP4 |  |  | 7643 | -0.210 | -0.3148 | No |
| 65 | SOX4 |  |  | 7958 | -0.268 | -0.3450 | No |
| 66 | UBE2C |  |  | 8278 | -0.348 | -0.3743 | No |
| 67 | CNP |  |  | 8294 | -0.352 | -0.3693 | No |
| 68 | HOMER3 |  |  | 8370 | -0.379 | -0.3705 | No |
| 69 | ENHO |  |  | 8432 | -0.400 | -0.3697 | No |
| 70 | NQO1 |  |  | 8588 | -0.479 | -0.3780 | No |
| 71 | PABPC1 |  |  | 8598 | -0.488 | -0.3698 | No |
| 72 | NDUFC1 |  |  | 8616 | -0.503 | -0.3621 | No |
| 73 | EEF2 |  |  | 8670 | -0.540 | -0.3578 | No |
| 74 | CDKN1A |  |  | 8679 | -0.545 | -0.3483 | No |
| 75 | ZFAS1 |  |  | 8753 | -0.615 | -0.3448 | No |
| 76 | RPL37 |  |  | 8808 | -0.656 | -0.3383 | No |
| 77 | RAB31 |  |  | 8831 | -0.687 | -0.3277 | No |
| 78 | RPL10 |  |  | 8835 | -0.690 | -0.3149 | No |
| 79 | RPL37A |  |  | 8860 | -0.721 | -0.3039 | No |
| 80 | RPS27 |  |  | 8865 | -0.727 | -0.2904 | No |
| 81 | ANXA2 |  |  | 8868 | -0.729 | -0.2768 | No |
| 82 | RPL22 |  |  | 8874 | -0.739 | -0.2633 | No |
| 83 | C9orf16 |  |  | 8876 | -0.744 | -0.2492 | No |
| 84 | RPL34 |  |  | 8877 | -0.744 | -0.2350 | No |
| 85 | RPL11 |  |  | 8899 | -0.771 | -0.2227 | No |
| 86 | PPP1R14B |  |  | 8900 | -0.771 | -0.2080 | No |
| 87 | RPLP0 |  |  | 8904 | -0.777 | -0.1935 | No |
| 88 | RPS28 |  |  | 8906 | -0.778 | -0.1788 | No |
| 89 | RPL18 |  |  | 8907 | -0.780 | -0.1639 | No |
| 90 | RPL3 |  |  | 8916 | -0.803 | -0.1495 | No |
| 91 | RPLP2 |  |  | 8932 | -0.844 | -0.1351 | No |
| 92 | RPL30 |  |  | 8937 | -0.850 | -0.1194 | No |
| 93 | RPS19 |  |  | 8941 | -0.855 | -0.1034 | No |
| 94 | RPL6 |  |  | 8942 | -0.856 | -0.0871 | No |
| 95 | RPL13 |  |  | 8944 | -0.862 | -0.0708 | No |
| 96 | EEF1A1 |  |  | 8958 | -0.915 | -0.0548 | No |
| 97 | RPLP1 |  |  | 8968 | -0.958 | -0.0376 | No |
| 98 | RPL18A |  |  | 8969 | -0.964 | -0.0192 | No |
| 99 | TMSB10 |  |  | 8979 | -1.090 | 0.0006 | No |
Table: GSEA details [plain text format]

  

Fig 2: LARSSON\_STATE\_5: Random ES distribution      
 Gene set null distribution of ES for **LARSSON\_STATE\_5**
